# Supplementary material for: Dynalign II: common secondary structure prediction for RNA homologs with domain insertions
Source: Nucleic Acids Res. 2014 Nov 21;42(22):13939–48. doi: 10.1093/nar/gku1172 (PMC4267632; doi:10.1093/nar/gku1172)
Supplement: SUPPLEMENTARY DATA [file supp_gku1172_nar-02021-z-2014-File012.zip › manual/javadoc/RNAstructure_java_utilities/BorderBuilder.html]

BorderBuilder


JavaScript is disabled on your browser.


- Overview
- Package
- Class
- Tree
- Index
- Help

- Prev Class
- Next Class

- Frames
- No Frames

- All Classes

- Summary:
- Nested |
- Field |
- Constr |
- Method

- Detail:
- Field |
- Constr |
- Method


RNAstructure\_java\_utilities

## Class BorderBuilder

- java.lang.Object
- - RNAstructure\_java\_utilities.BorderBuilder

- All Implemented Interfaces:
  :   Serializable

  ---

    

  ```
  public class BorderBuilder
  extends Object
  implements Serializable
  ```

  A class that places a border type on a specified component type.

  See Also:
  :   Serialized Form

- - ### Field Summary

    Fields

    | Modifier and Type | Field and Description |
    | --- | --- |
    | `private static long` | `serialVersionUID` |
  - ### Constructor Summary

    Constructors

    Constructor and Description

    `BorderBuilder()`
  - ### Method Summary

    Methods

    | Modifier and Type | Method and Description |
    | --- | --- |
    | `void` | `makeBottomBorder(int gap, JComponent comp)` Create an empty bottom border on a component. |
    | `private void` | `makeEmptyBorder(String type, int gap, JComponent comp)` Create an empty border on a component. |
    | `void` | `makeEqualBorder(int gap, JComponent comp)` Create an empty equidistant border on a component. |
    | `void` | `makeLeftBorder(int gap, JComponent comp)` Create an empty left border on a component. |
    | `void` | `makeRightBorder(int gap, JComponent comp)` Create an empty right border on a component. |
    | `void` | `makeTitledBorder(String title, JComponent comp)` Create a padded titled border on a component. |
    | `void` | `makeTopBorder(int gap, JComponent comp)` Create an empty top border on a component. |

    - ### Methods inherited from class java.lang.Object

      `clone, equals, finalize, getClass, hashCode, notify, notifyAll, toString, wait, wait, wait`

- - ### Field Detail


    - #### serialVersionUID

      ```
      private static final long serialVersionUID
      ```

      See Also:
      :   Constant Field Values
  - ### Constructor Detail


    - #### BorderBuilder

      ```
      public BorderBuilder()
      ```
  - ### Method Detail


    - #### makeBottomBorder

      ```
      public void makeBottomBorder(int gap,
                          JComponent comp)
      ```

      Create an empty bottom border on a component.

      Parameters:
      :   `gap` - The gap in pixels.
      :   `comp` - The component on which to create the border.


    - #### makeEmptyBorder

      ```
      private void makeEmptyBorder(String type,
                         int gap,
                         JComponent comp)
      ```

      Create an empty border on a component.

      Parameters:
      :   `type` - The border type.
      :   `gap` - The gap in pixels.
      :   `comp` - The component on which to create the border.


    - #### makeEqualBorder

      ```
      public void makeEqualBorder(int gap,
                         JComponent comp)
      ```

      Create an empty equidistant border on a component.

      Parameters:
      :   `gap` - The gap in pixels.
      :   `comp` - The component on which to create the border.


    - #### makeLeftBorder

      ```
      public void makeLeftBorder(int gap,
                        JComponent comp)
      ```

      Create an empty left border on a component.

      Parameters:
      :   `gap` - The gap in pixels.
      :   `comp` - The component on which to create the border.


    - #### makeRightBorder

      ```
      public void makeRightBorder(int gap,
                         JComponent comp)
      ```

      Create an empty right border on a component.

      Parameters:
      :   `gap` - The gap in pixels.
      :   `comp` - The component on which to create the border.


    - #### makeTitledBorder

      ```
      public void makeTitledBorder(String title,
                          JComponent comp)
      ```

      Create a padded titled border on a component.

      Parameters:
      :   `title` - The title of the border.
      :   `comp` - The component on which to create the border.


    - #### makeTopBorder

      ```
      public void makeTopBorder(int gap,
                       JComponent comp)
      ```

      Create an empty top border on a component.

      Parameters:
      :   `gap` - The gap in pixels.
      :   `comp` - The component on which to create the border.


- Overview
- Package
- Class
- Tree
- Index
- Help

- Prev Class
- Next Class

- Frames
- No Frames

- All Classes

- Summary:
- Nested |
- Field |
- Constr |
- Method

- Detail:
- Field |
- Constr |
- Method
